# Synergistic Preservation of Mutton Using a Biodegradable Edible Coating Incorporating *Lallemantia iberica* Mucilage and Cell‐Free Supernatant of *Lactobacillus helveticus* C7303

**DOI:** 10.1002/fsn3.72296

**Published:** 2026-09-01

**Authors:** Zeinab Mousavi, Behrooz Alizadeh Behbahani, Hossein Jooyandeh, Morteza Taki, Alireza Vasiee

**Affiliations:** ^1^ Department of Food Science and Technology, Faculty of Animal Science and Food Technology Agricultural Sciences and Natural Resources University of Khuzestan Mollasani Iran; ^2^ Department of Agricultural Machinery and Mechanization Engineering, Faculty of Agricultural Engineering and Rural Development Agricultural Sciences and Natural Resources University of Khuzestan Mollasani Iran; ^3^ Department of Food Safety and Quality Control Research Institute of Food Science and Technology (RIFST) Mashhad Iran

**Keywords:** biodegradable coating, cell‐free supernatant, lactic acid strain and mutton

## Abstract

In this study, a biodegradable coating was formulated using *Lallemantia iberica* seed mucilage (LISM) and varying concentrations of cell‐free supernatant (CFS) (1% and 2%) obtained from the lactic acid bacterium *Lactobacillus helveticus* C7303. Mutton was subsequently coated via the dipping method. The primary objective was to evaluate the coating's efficacy in extending the storage duration and to monitor microbial, physicochemical, and sensory changes in the coated meat, applying a significance threshold of *p* < 0.05. Evaluation of total viable counts, psychrotrophic bacteria, fungi, and coliforms revealed that while microbial numbers generally increased over storage time across all samples, they were significantly reduced in the coated mutton as the concentration of the *L. helveticus* C7303 CFS was increased. Compared to uncoated controls, the coated samples exhibited the lowest levels of pH, peroxide value, thiobarbituric acid (TBA), and total volatile basic nitrogen (TVBN), indicating enhanced oxidative and proteolytic stability. Furthermore, the coating substantially prevented the deterioration of moisture and hardness. Sensory and color analyses confirmed that mutton treated with the highest concentration of the CFS showed the highest overall acceptability. This novel biodegradable coating, utilizing *L. iberica* mucilage and *L. helveticus* C7303 CFS, successfully preserved meat quality and extended shelf life at 4°C by inhibiting oxygen exchange and curbing the proliferation of pathogenic microorganisms.

## Graphical abstract

A biodegradable bioactive coating was developed using *Lallemantia iberica* seed mucilage enriched with 1% and 2% cell‐free supernatant (CFS) from *Lactobacillus helveticus* C7303 and applied to mutton by dipping the meat samples. During storage at 4°C, the coating effectively inhibited microbial growth, including total viable bacteria, psychrotrophic bacteria, fungi, and coliforms, with stronger effects observed at higher CFS concentrations. Compared with the control, the coated samples exhibited lower pH values, peroxide values, thiobarbituric acid (TBA) levels, and total volatile basic nitrogen (TVBN) levels, indicating improved oxidative and proteolytic stability. The coating also preserved moisture, hardness, color, and sensory quality. The 2% CFS coating showed the greatest protective effect and the highest overall acceptability, demonstrating its potential to extend the shelf life of mutton by limiting oxygen exchange and suppressing microbial proliferation. Open AI's DALL‐E model was used to generate the graphical abstract to visually represent the research concept.

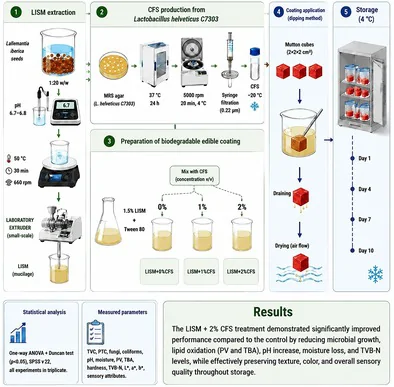

## 1 Introduction

Lipid oxidation is considered one of the most critical factors leading to the spoilage of mutton. This process is prevalent in mutton due to factors such as fermentable carbohydrates, high moisture content, high protein levels, an appropriate pH for microbial growth, and the presence of fatty acids with unsaturated bonds. Lipid oxidation is a major cause of tissue damage, quality deterioration, and reduced shelf life of mutton (Yan et al. 2024). Red meat contains substantial amounts of essential fatty acids, B vitamins, and minerals such as iron and zinc, playing a crucial role in human nutrition and health as a protein and nutrient source. Preserving meat quality while mitigating spoilage‐associated toxins and maintaining nutritional bioavailability remains a central challenge in food safety (Wu et al. 2025). Therefore, preserving its quality and nutritional value during storage until consumption is of significant concern.

Various preservation methods are employed to inhibit lipid oxidation and microbial growth in meat, including low‐temperature storage, the use of synthetic and chemical preservatives and antimicrobial compounds, vacuum packaging, and others. Currently, there is a global focus among societies and consumers on adopting novel preservation techniques that extend the shelf life of food products by inhibiting moisture and oxygen exchange and reducing microbial and enzymatic activity, while also being economically viable and posing no environmental hazard (Gong et al. 2024).

One such novel method is the use of edible and biodegradable coatings. These coatings incorporate bioactive compounds and natural antimicrobial agents which, besides being environmentally friendly, form protective, cohesive layers on the food surface. The gradual release of these antimicrobial agents and bioactive compounds helps to inhibit or reduce lipid oxidation and the growth of pathogenic microorganisms, thereby increasing the shelf life of the coated products (Liu et al. 2026).

Hydrocolloids, such as plant mucilages, play a prominent role in producing robust edible coatings (Barzegar et al. 2020). *Lallemantia iberica* (Balangu‐ye Shahri) is an annual plant recognized as an important source of mucilage. Its therapeutic properties have been utilized in traditional medicine for decades. Therefore, *L. iberica* mucilage is expected to not only provide structural strength but also enhance the antimicrobial performance of the edible coating (Noshad et al. 2020).

One of the bioactive substances used to boost the efficacy of edible coatings is the utilization of Generally Recognized As Safe (GRAS) lactic acid bacteria (LAB) (Alizadeh Behbahani et al. 2023; Li et al. 2022). LAB are capable of producing antimicrobial and antioxidant compounds, including organic acids, diacetyl, bacteriocins, and various enzymes (Alizadeh Behbahani et al. 2024). Consequently, incorporating LAB strains such as *Lactobacillus helveticus* will enhance coating performance by reducing the activity of various microorganisms, preventing lipid oxidation, and extending the shelf life of coated food products (Ozma et al. 2022).

Mousavi et al. (2026a) previously investigated the use of cell‐free supernatant (CFS) derived from *Lactobacillus helveticus* Lh 23 in a *Lallemantia iberica* seed mucilage (LISM) coating to improve the shelf life of lamb (Mousavi et al. 2026a). Building on that study, the present work employs CFS obtained from the indigenous *L. helveticus* C7303 strain in the same coating system for mutton preservation. Because the antimicrobial and other bioactive properties of CFS are strain‐dependent (Mousavi et al. 2026b), the findings obtained with CFS from Lh 23 cannot be directly extrapolated to CFS from C7303. Accordingly, by focusing on C7303 and using the same LISM matrix, the present study provides independent evidence for the performance of this system in mutton.

In the present study, an edible and biodegradable coating was prepared after the extraction of LISM and the CFS of the *L. helveticus* C7303. Mutton slices were coated and stored at 4°C for 1, 4, 7, and 10 days, after which the extended shelf life and the microbial, physicochemical, and sensory properties of the coated mutton were evaluated. The parameters assessed in the meat samples included: Total Viable Count (TVC), Psychrotrophic Count (PTC), fungi and coliform counts, changes in pH, moisture, peroxide value, thiobarbituric acid (TBA) content, hardness, total volatile basic nitrogen (TVBN), color parameters (L*, a*, b*), and sensory attributes (odor, appearance, texture, and overall acceptability).

## 2 Materials and Methods

### 2.1 Extraction of Mucilage From the *Lallemantia iberica* Plant

The mucilage was extracted following the procedures outlined by Noshad et al. (2020) and Alizadeh Behbahani et al. (2024). The plant seeds were sourced from a local market in Ahvaz city (Khuzestan province), and their scientific identification was verified before the necessary extraction steps commenced. The seeds of *Lallemantia iberica* were combined with deionized water at a 1:20 ratio, and the mucilage was extracted at a temperature of 50°C and a pH range of 6.7–6.8. To ensure the complete separation of the mucilage from the seed surfaces, a rotary plate extractor was employed.

### 2.2 Preparation of CFS of *L. helveticus* Strain C7303

The CFS from the *L. helveticus* C7303 was prepared using the method described by Barzegar et al. (2024) (Mousavi et al. 2026b). The strain was cultured in De Man, Rogosa and Sharpe (MRS) agar medium. After incubation for 24 h at 37°C, the culture was centrifuged at 5000 rpm for 20 min at 4°C. Subsequently, a syringe filter was used to filter the supernatant for CFS collection. Following extraction, the CFS was stored at −20°C.

### 2.3 Preparation of Edible Coating and Coating of Mutton

Following the method described by Alizadeh Behbahani et al. (2024). the coating solution was prepared. In this study, fresh mutton (approximately 6 months of age) was used. Mutton samples were cut into uniform cubes of 2 × 2 × 2 cm^3^ and used for the experiments. First, a 1.5% LISM solution was prepared in sterile distilled water containing a specified amount of Tween 80. Then, to formulate the active edible coating, CFS was added to the mucilage solution at three different concentrations (0%, 1%, and 2%). Accordingly, four treatments were considered: Control (without coating), LISM +0% CFS, LISM +1% CFS, and LISM +2.0% CFS. The mutton cubes were coated by the dipping method using the respective coating solutions. After drying at room temperature, the coated samples were stored in a refrigerator at 4°C. Analyses were carried out during the storage period on days 1, 4, 7, and 10.

### 2.4 Microbial Enumeration of Coated Mutton Samples

Microbial analysis was conducted according to the method described by Tanavar et al. (2021). For the growth and enumeration of the TVC, Plate Count Agar (PCA) medium was used with incubation at 37°C for 48 h. To quantify PTC, PCA medium was employed, followed by incubation at 7°C for 10 days. Fungi were counted using Sabouraud Dextrose Agar (SDA) medium and incubated at 27°C for 72 h. Finally, the enumeration of coliforms utilized Violet Red Bile Agar (VRBA) medium, with incubation at 37°C for 24 h. All resulting counts were reported as log Colony Forming Unit/g (log CFU/g).

### 2.5 Evaluation of pH Changes in Coated Mutton Samples

The pH of the meat samples was evaluated using a calibrated pH meter, following the method described by Rouhi et al. (2024). For the measurement, a 5 g portion of the sample was homogenized with 45 mL of sterile distilled water, and the final pH of the resulting mixture was then measured.

### 2.6 Evaluation of Moisture Changes in Coated Mutton Samples

The moisture content of the samples was determined according to the method established by Malvano et al. (2022). The samples were dried in a hot air oven at 102°C for 4 to 6 h. The moisture content was then calculated using Equation (1) and reported as a percentage.

(1) $$\begin{array}{c} \text{Moisture}\;\left(\%\right)=100\\ \;\times \left(\left(\text{initial sample weight}-\text{final sample weight}\right)/\left(\text{initial sample weight}\right)\right) \end{array}$$

### 2.7 Evaluation of Peroxide Content of Coated Mutton Samples

This assay was performed according to the method described by Saffari Samani et al. (2023). The fat from the coated mutton samples was first extracted using a chloroform‐acetic acid solution. To liberate the iodine present in the sample, 0.5 mL of saturated potassium iodide, 30 mL of distilled water, and 0.5 mL of a 1% starch solution were added to the extracted fat. The mixture was then titrated with 0.01 N sodium thiosulfate solution. The results were reported in terms of meq O_2_ per kg (meq O_2_/kg) of fat.

### 2.8 Evaluation of the Amount of TBA in Coated Mutton Samples

This assay was performed according to the method described by Lashkari et al. (2020). Initially, 2 g of the sample was mixed with 5 mL of 20% trichloroacetic acid solution and 5 mL of 0.01 M TBA solution. This mixture was then heated at 100°C. After measuring the absorbance of the samples at a wavelength of 532 nm, the results were reported in terms of milligrams of malondialdehyde per kilogram (mg MDA/kg).

### 2.9 Evaluation of the Hardness of Coated Mutton Samples

This assay was conducted according to the method detailed by Alizadeh Behbahani et al. (2021). The meat samples were compressed using a cylindrical aluminum probe with a diameter of 36 mm at a test speed of 5 mm/s. The maximum force (N) exerted during this compression was recorded and defined as the hardness value.

### 2.10 Evaluation of TVB‐N in Coated Mutton Samples

This assay was performed according to the method described by Goulas and Kontominas (2005). A 10 g sample of the meat was mixed with 50 mL of sterile distilled water, 2 g of Magnesium Oxide (MgO), and one drop of silicon (likely antifoam). A mixture of 25 mL of 3% boric acid and 0.04 mL of the indicator dyes methyl red and methylene blue was prepared for titration collection. Titration with 0.1 N hydrochloric acid (HCl) commenced once the color of the collecting solution turned green. The titration continued until a pink color persisted upon the addition of one final drop of 0.1 N HCl. The TVB‐N content was calculated and reported in mg/100 g based on the following Equation (2):

(2) $$\mathrm{TVB}-N\;\left(\mathrm{mg}/100\;g\right)=\left(V\times C\times 14\times 100\right)/10$$

Were, *V* is the volume and *C* is the concentration (0.1 N) of the consumed hydrochloric acid.

### 2.11 Color Evaluation of Coated Mutton Samples

According to the method of Faradonbeh et al. (2024), the lightness (L*), yellowness (b*) and redness (a*) indices were measured using a colorimeter (Faradonbeh et al. 2024).

### 2.12 Sensory Evaluation of Coated Mutton Samples

This sensory evaluation was conducted in full accordance with research ethical guidelines, and the sensory evaluation protocol of this study was reviewed and approved by the Research Ethics Committee of the Agricultural Sciences and Natural Resources University of Khuzestan, Iran. Prior to participation, the purpose and procedures of the experiment were fully explained to all participants, and informed consent was obtained from all assessors. Throughout the study, the safety and well‐being of the volunteers were prioritized, potential risks were minimized, and the anonymity of participants and confidentiality of data were fully ensured. The information obtained was used solely for scientific purposes. The sensory evaluation was carried out by a panel of 52 semi‐trained assessors, including 28 men and 24 women, aged 25 to 40 years. At each storage interval (Days 1, 4, 7, and 10), samples from three independent production replicates were presented to the panel in random order. Raw mutton samples were cut into uniform pieces (approximately 2 × 2 × 2 cm) and presented at ambient temperature. The evaluation was performed in a well‐lit, odor‐free room using a blind design, in which the samples were labeled with randomized three‐digit codes to prevent bias. The assessors evaluated the samples for color, odor, texture, and overall acceptability using a 9‐point hedonic scale (1 = dislike extremely to 9 = like extremely). To prevent sensory fatigue, the assessors were instructed to rest between the evaluation of each sample (Noshad et al. 2021).

### 2.13 Statistical Data Analysis

To evaluate the obtained data, one‐way analysis of variance (ANOVA) and Duncan's multiple range test were employed using the SPSS software (version 22) at a 5% significance level (*p* < 0.05). To ensure accuracy, all experiments were conducted in triplicate.

## 3 Results and Discussion

**FIGURE 1:**
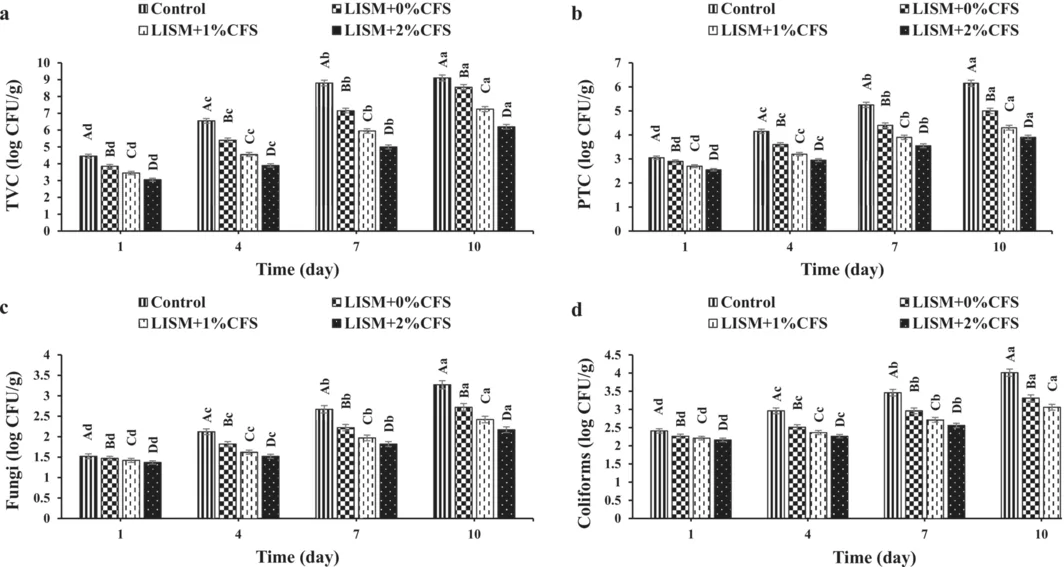
Changes in total viable count (a), psychrotrophic count (b), fungal count (c), and coliform count (d) of mutton samples coated with *Lallemantia iberica* seed mucilage (LISM) and different levels of cell‐free supernatant (CFS) of *Lactobacillus* helveticus strain C7303 during storage. Mean value ± standard deviation is presented. Capital letters indicate significant differences among treatments (A–D), and lowercase letters indicate significant differences across storage days (a–d) (*p* < 0.05) (*n* = 3 independent replicates).

In the present study, the counts of TVC, PTC, molds/yeasts, and coliforms were evaluated in uncovered mutton meat samples control (without coating), LISM +0% CFS, LISM +1% CFS, and LISM +2.0% CFS during refrigerated storage at 4°C on Days 1, 4, 7, and 10. As shown in Figure 1, the minimum and maximum microbial loads were recorded on Days 1 and 10 of storage, respectively (*p* < 0.05). According to the TVC and PTC assessments (Figure 1a,b), significant differences were observed among the samples on days 4, 7, and 10 (*p* < 0.05). The International Commission on Microbiological Specifications for Foods (ICMSF) has set 7.0 log CFU/g as the maximum acceptable limit for TVC in red meat (Hernández et al. 2009). Furthermore, increasing the concentration of CFS obtained from *L. helveticus* C7303 in LISM coatings (LISM +1.0% and LISM +2.0% samples) significantly reduced the growth and proliferation of microorganisms (*p* < 0.05). This marked reduction is the result of a strong synergistic effect, in which the mucilage polymer matrix acts both as a physical barrier against secondary contamination and as a medium enabling the entrapment and sustained release of bioactive compounds present in the CFS onto the meat surface (Namazi et al. 2025; Mirmoeini et al. 2025). In addition, the potent antimicrobial activity of the CFS derived from *L. helveticus* C7303 plays a key role through the production of antimicrobial peptides, organic acids (such as lactic acid), and various enzymes, components that collectively inhibit the growth of diverse microbial groups. Notably, the organic acids in the CFS reduce the surface pH of the meat and penetrate bacterial cell membranes, disrupting the electrochemical gradient and leading to cell death.

Based on Figure 1c,d, the results for molds/yeasts and coliforms showed significant differences among all samples during storage (*p* < 0.05), except on day 1 when no significant differences were observed among the coated samples (*p* > 0.05). The observed reduction in mold/yeast counts and aerobic psychrotrophic bacteria such as *Pseudomonas* in coated samples is attributed to the limited oxygen diffusion caused by the biodegradable films (Do et al. 2024). Beyond the oxygen barrier effect, the marked reduction in coliforms (as Gram‐negative bacteria) in the CFS‐containing samples is particularly noteworthy. Although bacteriocins produced by lactic acid bacteria typically exhibit stronger activity against Gram‐positive bacteria, the organic acids present in the CFS can increase the permeability of the outer membrane of Gram‐negative bacteria, thereby facilitating bacteriocin entry and subsequent cellular disruption (Namazi et al. 2026b; Soundrarajan et al. 2026). El‐Mokhtar et al. (2020) specifically reported that the antimicrobial activity of CFS produced by lactic acid bacteria is primarily associated with the presence of bioactive antimicrobial compounds such as bacteriocins (Do et al. 2024). In a study similar to the present investigation, a biodegradable coating based on *Malva sylvestris* mucilage combined with *L. paracasei* ‐derived postbiotics successfully maintained microbial quality and reduced microbial loads in coated mutton meat samples during storage, compared to the control (Ozma et al. 2022). These findings are consistent with the results of the current study. Overall, the results demonstrate that combining plant‐derived mucilage with LAB‐derived postbiotics can significantly slow the microbial spoilage of fresh meat and extend its shelf life without the need for synthetic preservatives.

**FIGURE 2:**
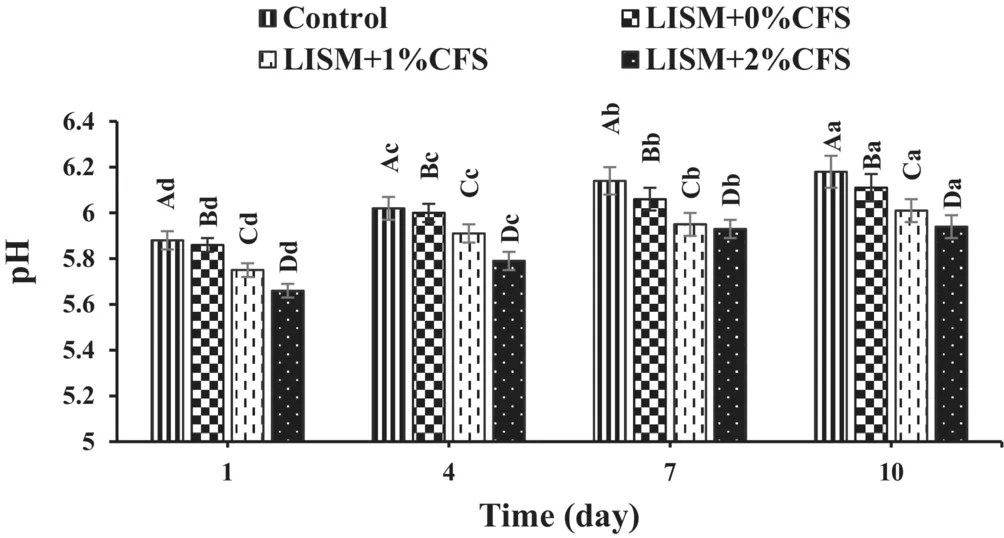
pH changes of mutton samples coated with *Lallemantia iberica* seed mucilage (LISM) and different levels of cell‐free supernatant (CFS) of *Lactobacillus helveticus* strain C7303 during storage. Mean value ± standard deviation is presented. Capital letters indicate significant differences among treatments (A–D), and lowercase letters indicate significant differences across storage days (a–d) (*p* < 0.05), (*n = 3 in* dependent replicates).

As shown in Figure 2, the pH changes in mutton meat samples coated with LISM and the CFS derived from *L. helveticus* C7303 were examined. The results indicated that the pH of all samples increased with prolonged storage. This gradual rise in pH during meat storage is a common phenomenon associated with the spoilage process and is primarily attributed to the activity of spoilage microorganisms and the degradation of protein compounds. In this process, proteolytic enzymes produced by microorganisms break down muscle proteins, generating alkaline compounds such as amines and ammonia, which ultimately lead to pH elevation. Therefore, samples with a higher microbial load typically exhibit a more pronounced increase in pH (Namazi et al. 2026a). Consistent with the findings of the present study, microbial activity in meat samples increased with extended storage duration. The microbial activity, specifically the production of degrading enzymes like microbial proteases, is detrimental as these proteases hydrolyze proteins, leading to the release of basic compounds and a consequent increase in the pH of the meat samples (Zhong et al. 2024). In a study analogous to the current research, Abbasi et al. (2023) reported that the antimicrobial effect of CFS from the *Saccharomyces cerevisiae var. boulardii* probiotic yeast strain preserved collagen and myofibrillar proteins during the storage of coated meat samples (Abbasi et al. 2023). However, the evaluation results presented in Figure 2 showed that significant differences were observed among the samples across all storage periods (*p* < 0.05), and the coating based on LISM and CFS effectively inhibited the upward trend of pH (*p* < 0.05). Notably, the lowest pH value after 10 days of storage belonged to the samples coated with mucilage and 2% CFS. This controlled suppression of pH increase in the treated samples can be attributed to the antimicrobial activity of the bioactive compounds present in the CFS. These compounds inhibit the growth and metabolic activity of spoilage microorganisms, thereby reducing proteolytic reactions and limiting the formation of alkaline compounds. Moreover, the reduction in microbial activity in the presence of CFS may contribute to better preservation of collagen and myofibrillar proteins during cold storage, an effect that plays a pivotal role in maintaining the chemical stability and overall quality of the meat (Abbasi et al. 2024; Noshad et al. 2026). A study investigated the effect of a bioactive coating based on *Lepidium perfoliatum* seed mucilage (LPSM) and the CFS of *L. helveticus* on the shelf life of beef. The results of that study showed that samples coated with LPSM combined with 2% CFS effectively slowed the microbial spoilage process and extended the meat's shelf life by maintaining the lowest pH values during the storage period (Namazi et al. 2026a). Furthermore, Jiang et al. (2024) stated that the establishment of lactobacilli on the meat surface and their production of acid is the main reason for the pH reduction in fresh meat (Jiang et al. 2024).

**FIGURE 3:**
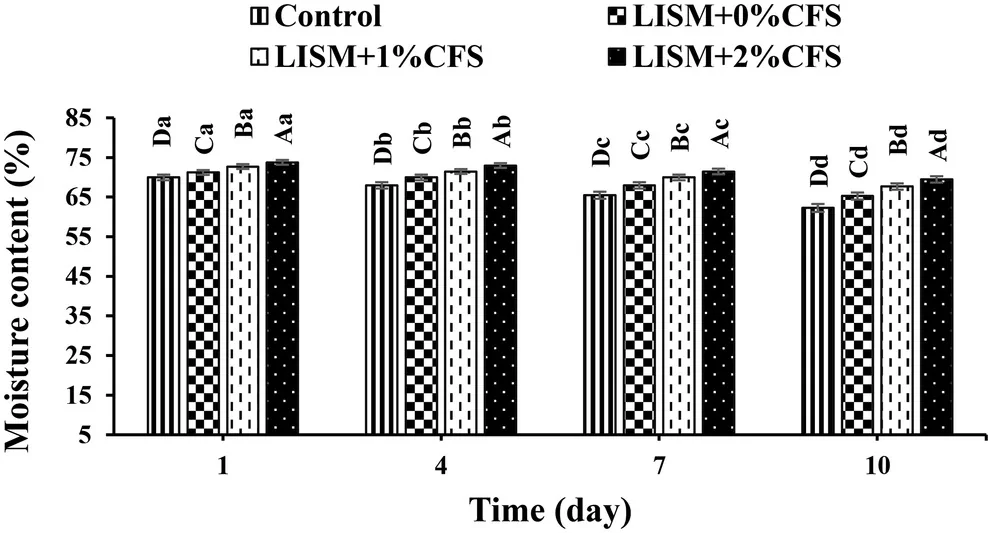
Moisture content changes of mutton samples coated with *Lallemantia iberica* seed mucilage (LISM) and different levels of cell‐free supernatant (CFS) of *Lactobacillus helveticus* strain C7303 during storage. Mean value ± standard deviation is presented. Capital letters indicate significant differences among treatments (A–D), and lowercase letters indicate significant differences across storage days (a–d) (*p* < 0.05), (*n = 3 in* dependent replicates).

The changes in moisture content of mutton meat samples coated with LISM mucilage and CFS from the *L. helveticus* C7303 strain are shown in Figure 3. The results indicated a downward trend in moisture content for all samples over time, with the highest moisture recorded on the first day and the lowest at the end of the storage period (Day 10). However, the application of the Balangu mucilage‐based coating, especially in combination with CFS, effectively prevented severe moisture loss (*p* < 0.05). On the 10th day, the control sample experienced a significant drop, reaching the lowest moisture content, while the sample coated with the highest concentration (LISM +2.0% CFS) managed to retain the highest moisture level among all treatments. Statistical analysis confirmed a significant difference (*p* < 0.05) between the coated samples and the control throughout all storage days. Biodegradable coatings based on mucilages form resistant protective layers on the surface of food products by creating hydrogen bonds, thereby effectively preventing moisture loss in coated samples (Liu et al. 2026). Meanwhile, the CFS derived from LAB, by distributing uniformly within the mucilage matrix, creates a cohesive network inside the coating that also restricts the movement of water molecules (Ma et al. 2020; Ayadi et al. 2026). Consistent with the findings of the present study, Barzegar et al. (2024) reported that the lowest moisture loss in coated sheep meat samples was due to the presence of bioactive compounds in the coating. Alizadeh Behbahani et al. (2024) stated that a coating based on *Plantago major* mucilage and the CFS of the probiotic bacterium *Lactococcus lactis* NJ414 effectively prevented moisture loss from meat samples during the storage period and maintained a higher moisture content compared to the control group (Alizadeh Behbahani et al. 2024).

Another adverse consequence of increased microbial activity in meat is the acceleration of lipid oxidation due to microbial enzymes and the subsequent formation of its secondary products. These secondary compounds cause off‐flavors and off‐odors, severely degrading the quality of the meat. The process of lipid oxidation, by producing short‐chain fatty acids, leads to an increase in the PV and TBA indices in the samples (Hai et al. 2021; Mukherjee et al. 2026). According to reference quality standards, the maximum permissible limit for the PV index in meat is defined as 7.0 meq O_2_/kg, and the highest acceptable limit for the TBA index is 1.0 mg MDA/kg (Rouhi et al. 2024). To evaluate the extent of lipid oxidation, the PV of the samples was measured during the storage period (Table 1). The results indicated that this index increased in all treatments over time, demonstrating the progression of the oxidation process. However, the coated samples, particularly those containing different concentrations of CFS, showed significantly lower PV values compared to the control sample (*p* < 0.05). This effective trend was evident from the first day of storage, as the PV in the control sample (2.71 ± 0.15 meq O_2_/kg) was significantly higher than in the LISM+2% CFS sample (1.41 ± 0.11 meq O_2_/kg). This difference was intensified on the tenth day of storage, where the control sample reached the highest PV of 14.36 ± 0.30 meq O_2_/kg (far exceeding the standard permissible limit), while the LISM+2% CFS treatment, with a value of 6.26 ± 0.20 meq O_2_/kg, exhibited the lowest oxidation and remained within the acceptable range. The increase in the PV in the control sample is attributed to the direct oxidation of lipids in the absence of a physical barrier against oxygen, as well as the activity of oxidative enzymes (such as lipases) produced by the uncontrolled microflora on the meat surface (Najar et al. 2024). In contrast, the inhibition of oxidation in the treated samples is due to the activity of potent antioxidant compounds present in the CFS from the *L. helveticus* C7303. These metabolites, including bioactive peptides and organic acids, act through two main pathways: chelating pro‐oxidant metal ions (such as Fe^2+^) and scavenging free radicals (Namazi et al. 2026a; Razavi et al. 2026). Consistent with the results of the present study, Jooyandeh kar et al. demonstrated that the application of a combined coating of *Alyssum homolocarpum* seed mucilage and CFS derived from *Lactiplantibacillus pentosus* SM1 had a significant effect on reducing oxidation. Specifically, the PV for the uncoated (control) samples and the samples coated with AHSM +2 CFS were reported as 5.92 and 2.71 meq O_2_/kg, respectively (Jooyandeh Kar et al. 2026). Furthermore, in a similar study, Mirzaei et al. (2025) investigated a coating based on mucilage and CFS obtained from probiotic. Their findings also indicated a significant decrease in the peroxide value of the coated samples compared to the control group (Mirzaei et al. 2025).

**TABLE 1:** Effect of *Lallemantia iberica* seed mucilage (LISM) coating incorporated with different levels of cell‐free supernatant (CFS) from *Lactobacillus helveticus* strain C7303 on the peroxide value (PV), thiobarbituric acid (TBA), and total volatile basic nitrogen (TVB‐N) of mutton samples during storage.

Parameter	Storage days	Treatments			
		Control	LISM+0%CFS	LISM+1%CFS	LISM+2%CFS
Peroxide (meq O_2_/kg)	1	2.71 ± 0.15^Ad^	2.26 ± 0.13^Bd^	1.81 ± 0.12^Cd^	1.41 ± 0.11^Dd^
	4	6.21 ± 0.20^Ac^	4.61 ± 0.17^Bc^	3.41 ± 0.16^Cc^	2.61 ± 0.14^Dc^
	7	9.90 ± 0.24^Ab^	7.10 ± 0.20^Bb^	5.45 ± 0.18^Cb^	4.20 ± 0.17^Db^
	10	14.36 ± 0.30^Aa^	9.86 ± 0.24^Ba^	8.01 ± 0.22^Ca^	6.26 ± 0.20^Da^
TBA (mg MDA/kg)	1	0.48 ± 0.03^Ad^	0.42 ± 0.03^Bd^	0.34 ± 0.02^Cd^	0.28 ± 0.02^Dd^
	4	1.01 ± 0.05^Ac^	0.92 ± 0.04^Bc^	0.65 ± 0.04^Cc^	0.50 ± 0.03^Dc^
	7	1.19 ± 0.07^Ab^	1.10 ± 0.04^Bb^	0.92 ± 0.05^Cb^	0.78 ± 0.04^Db^
	10	1.29 ± 0.09^Aa^	1.16 ± 0.07^Ba^	0.97 ± 0.06^Ca^	0.91 ± 0.05^Da^
TVB‐N (mg/100 g)	1	5.56 ± 0.45^Ad^	4.76 ± 0.40^Bd^	4.06 ± 0.38^Cd^	3.56 ± 0.35^Dd^
	4	14.95 ± 0.55^Ac^	10.45 ± 0.50^Bc^	8.65 ± 0.48^Cc^	7.35 ± 0.42^Dc^
	7	25.56 ± 0.62^Ab^	18.10 ± 0.56^Bb^	14.60 ± 0.52^Cb^	12.10 ± 0.48^Db^
	10	30.06 ± 0.75^Aa^	25.56 ± 0.65^Ba^	20.56 ± 0.60^Ca^	17.26 ± 0.55^Da^

*Note:* Mean value ± standard deviation is presented. Capital letters indicate significant differences among treatments (A–D), and lowercase letters indicate significant differences across storage days (a–d) (*p* < 0.05) (*n* = 3 independent replicates).

The results of the TBA index, a measure of secondary lipid oxidation, are presented in Table 1 The increase in the TBA index, which indicates the production of malondialdehyde and other aldehydes from the decomposition of hydroperoxides, is directly related to the unpleasant taste and smell of rancidity in meat (Kamankesh et al. 2015; Al‐Qadiri et al. 2025). Similar to the peroxide value, TBA values in all samples increased throughout the storage period (*p* < 0.05). On the first day, the control sample had the highest TBA value (0.48 ± 0.03 mg MDA/kg), whereas the LISM +2% CFS treatment (0.28 ± 0.02 mg MDA/kg) demonstrated the immediate effectiveness of the coating in controlling oxidation. A key milestone was observed on the fourth day when the control sample surpassed the standard acceptable limit of 1.0 mg MDA/kg (Rouhi et al. 2024), rendering the meat's sensory‐chemical quality unacceptable for consumers. In contrast, the fact that the LISM +2% CFS treatment remained within the acceptable range until after day seven directly translates to an extension of the product's shelf‐life. The steep upward trend of TBA in the control sample indicates an uncontrolled oxidation chain, where primary oxidation products (measured by PV) are rapidly converted into toxic and undesirable secondary products (Karimi et al. 2025). These results fully confirm and complement the findings for the PV. At the end of the storage period (day 10), the control sample reached the highest level of oxidation (1.29 ± 0.09 mg MDA/kg). In contrast, the coatings containing CFS effectively inhibited this process, with values of 0.97 ± 0.06 (for LISM +1% CFS) and 0.91 ± 0.05 mg MDA/kg (for LISM +2% CFS). The success of this bioactive coating is rooted in a dual defense mechanism: the *Lallemantia iberica* mucilage acts as a physical barrier against oxygen, and the active metabolites in the CFS inhibit this destructive process at a biochemical level by neutralizing free radicals (Hashemi et al. 2023). In the study by Zanganeh et al. (2023), the effect of an edible coating based on LISM containing grapefruit essential oil nanoemulsion (CPN) was evaluated. The investigation of lipid oxidation indices showed that after 9 days of cold storage, the level in the sample coated with LISM+2% CPN significantly decreased to 0.59 mg MDA/kg, whereas this value was 1.20 mg MDA/kg for the control sample (Zanganeh et al. 2023). In the study by Rahman et al. (2025), the use of postbiotics derived from LA ( *Lactobacillus acidophilus* ) and LGG (*Lacticaseibacillus rhamnosus GG*) reduced the lipid oxidation (TBARS index) of chicken meat from 0.82 (in the control sample) to 0.67 mg MDA/kg. Among them, the LGG postbiotic demonstrated a stronger antioxidant effect in preventing lipid oxidation (Rahman et al. 2025). While in a similar study, the application of mucilage coating containing 2% CFS obtained from probiotic on mutton resulted in a significant reduction in TBA values compared to the control (Mirzaei et al. 2025).

TVB‐N is one of the most important biochemical criteria and vital indicators for evaluating the extent of protein spoilage and the quality of red meat caused by microbial and enzymatic activities, with an acceptable limit of 25 mg/100 g (Alshehry 2025). As the results presented in Table 1 show, the trend of changes in this index was upward in all treatments during the storage period. On the first day, the TVB‐N value in the control sample was 5.56 ± 0.45 mg/100 g, which was significantly (*p* < 0.05) higher than the other coated treatments. Over time, on days 4 and 7, this difference became more pronounced, and the control sample progressed towards high spoilage values with a steeper slope. At the end of the storage period (day 10), the treatments containing LISM coating combined with 1% and 2% CFS recorded values of 20.56 ± 0.60 and 17.26 ± 0.55 mg/100 g, respectively, demonstrating a highly significant decrease compared to the control sample (30.06 ± 0.75 mg/100 g) (*p* < 0.05). Based on these results, while the control sample exceeded the permissible spoilage limit, the LISM+2%CFS treatment exhibited the best performance in inhibiting protein spoilage and delaying the deterioration of product quality by maintaining the lowest TVB‐N level throughout the evaluation period. In fact, preventing the growth of various microorganisms, especially *Pseudomonas* species, and inhibiting the lipid oxidation process are essential to prevent an excessive increase of TVB‐N in red meat; the results indicate that this bioactive coating was able to play an effective role in controlling this trend (Yousefi et al. 2018). In explaining this efficacy, the reduced accumulation of nitrogenous metabolites such as ammonia and biogenic amines in the optimal treatment is directly related to the inhibition of bacterial decarboxylase and deaminase activities by organic acids, bacteriocins, and the localized pH drop caused by the postbiotic compounds of CFS. In this regard, the mucilage polymer network, in addition to the controlled release of these compounds, significantly suppresses the oxidative deamination rate of amino acids in the muscle tissue by creating a low‐oxygen microenvironment, thereby preventing the degradation of myofibrillar proteins (Wook et al. 2016). Barzegar et al. (2023) reported that a bioactive coating based on Shirazi Balangu seed mucilage containing CFS from *Levilactobacillus brevis* G145 successfully inhibited the growth of microbial pathogens (including TVC, PTC, molds, *Staphylococcus aureus* , and *E. coli* ) and consequently prevented the excessive increase of TVB‐N in coated lamb samples. The results of this study are in agreement with the findings of Alizadeh Behbahani et al. (2024) and Barzegar et al. (2024).

**FIGURE 4:**
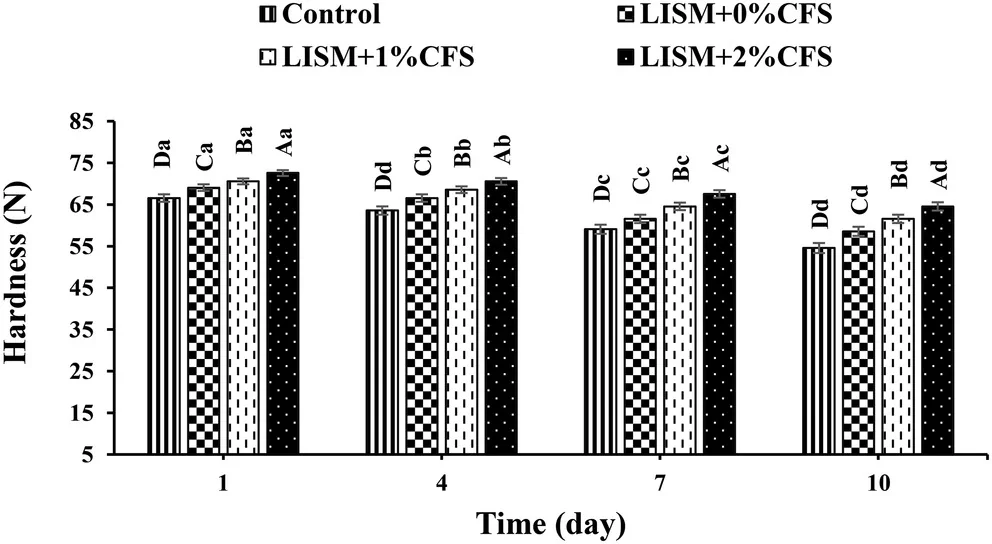
Changes in the hardness value of mutton samples coated with *Lallemantia iberica* seed mucilage (LISM) and different levels of cell‐free supernatant (CFS) of *Lactobacillus helveticus* strain C7303 during storage. Mean value ± standard deviation is presented. Capital letters indicate significant differences among treatments (A–D), and lowercase letters indicate significant differences across storage days (a–d) (*p* < 0.05), (*n* = 3 in dependent replicates).

Meat texture and hardness is one of the most important physical indicators in evaluating quality, maintaining structural integrity, and consumer acceptability, the decline of which is directly related to the degradation of myofibrillar proteins by enzymatic and microbial activities during the storage period (Mousavi et al. 2026a). The results regarding the trend of changes in the tissue hardness index of meat samples during the storage period are presented in Figure 4. On the first day, the hardness value in the control sample was 66.56 N, which was significantly (*p* < 0.05) lower than the coated treatments. Over time and upon reaching the end of the storage period (day 10), the hardness of the control sample experienced structural decline at a steeper rate and dropped to 54.56 N. In contrast, the treatments with LISM coating enriched with 1% and 2% CFS successfully recorded values of 61.56 and 65.56 N on the tenth day, respectively, indicating a significant preservation of texture compared to the control sample (*p* < 0.05). Based on these findings, the LISM+2% CFS treatment, by maintaining the highest level of the hardness index on all evaluation days, demonstrated the best performance in delaying the meat tissue softening process. This effectiveness can be attributed to the protective role of the polymeric network and the bioactive compounds of CFS in inhibiting the growth of spoilage microorganisms and reducing the activity rate of proteolytic (protein‐degrading) enzymes, which altogether prevent the premature collapse of the meat tissue network (Duran and Kahve 2020; Ghodsi Sheykhjan et al. 2025). The escalation of microbial activity and the production of destructive enzymes cause multiple tissue damages, such as collagen degradation and myofibrillar protein breakdown, resulting in a reduction in meat hardness (Barzegar et al. 2020). In the present study, the results for hardness were consistent with the microbial evaluation, and these findings align with the conclusions of other researchers (Noshad et al. 2020; Abbasi et al. 2023). In a study, Noshad et al. (2026) demonstrated that the use of a biodegradable coating based on Qodoume Shahri mucilage and the postbiotic *Limosilactobacillus reuteri* on beef effectively prevented the decrease in meat texture hardness during storage and maintained its physical structure by inhibiting the activity of proteolytic enzymes and preventing protein degradation (Noshad et al. 2026). These findings are also consistent with our previous study, in which the use of *Lallemantia iberica* mucilage and 2% CFS from *L. helveticus* LH 23 in lamb meat on the final day resulted in a hardness value of 64.00 N (Mousavi et al. 2026a).

**FIGURE 5:**
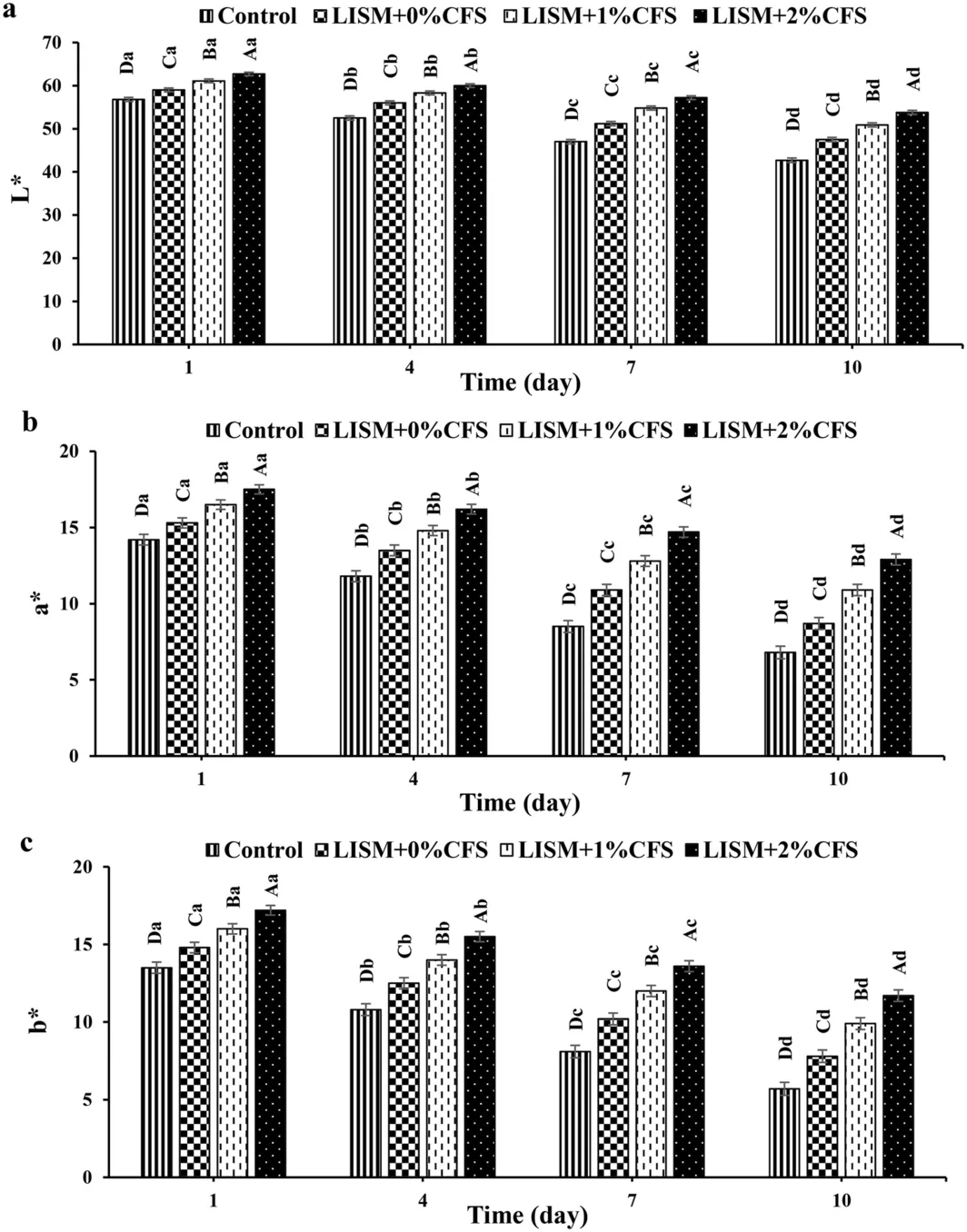
The changes in L* (a), a* (b), b* (c) value of mutton samples coated with *Lallemantia iberica* seed mucilage (LISM) and different levels of cell‐free supernatant (CFS) of *Lactobacillus helveticus* strain C7303 during storage. Mean value ± standard deviation is presented. Capital letters indicate significant differences among treatments (A–D), and lowercase letters indicate significant differences across storage days (a–d) (*p* < 0.05) (*n* = 3 independent replicates).

Meat color is one of the most important visual indicators in evaluating quality, freshness, and determining consumer acceptance. The results evaluating the trend of changes in the color parameters (L* for lightness, a* for redness, and b* for yellowness) of the meat samples during 10 days of storage are presented in Figure 5. Examination of the data indicates that the levels of all three color parameters in all experimental groups exhibited a downward trend over time; however, the slope of this decline was much gentler in the coated samples. By the end of the storage period (day 10), the L* in the control sample experienced a sharp drop to 42.70 ± 0.52, whereas this value was maintained at its highest level, namely 53.8 ± 0.46, in the LISM+2% CFS treatment (*p* < 0.05). Similarly, the a*, which is the most vital factor in perceiving meat freshness, decreased to a minimum value of 6.80 ± 0.41 for the control sample on the tenth day. In contrast, the LISM+2% CFS treatment successfully preserved this index at a remarkable value of 12.90 ± 0.36, showing a significant difference from the control group (*p* < 0.05). This trend was also repeated for the b*; the lowest rate of decline was specifically observed in the LISM+2% CFS treatment, which recorded a value of 11.70 ± 0.37 on the tenth day (*p* < 0.05). Based on these findings, the LISM+2% CFS treatment demonstrated the best performance in preserving all three color parameters. The decline of color indices during storage generally occurs due to the oxidation of meat pigments; as the decrease in oxy‐myoglobin due to oxidation leads to the formation of met‐myoglobin and a consequent reduction in L*, a*, and b* values in red meat (Hoa et al. 2024). In this regard, impermeability to oxygen and the reduced rate of oxy‐myoglobin oxidation are important factors for color stability, and incorporating bioactive compounds into coatings provides a desirable protective effect against color changes (Alexandre et al. 2021). Accordingly, the success of the superior treatment can be attributed to the protective role of the coating's physical barrier in reducing oxygen permeation, as well as the high antioxidant potential of its bioactive compounds, which effectively prevent pigment oxidation and met‐myoglobin formation, ensuring the color stability of the product throughout the storage period (Majdi et al. 2024; Noshad et al. 2022). The results of the current study are consistent with the findings of Abbasi et al. (2024), who reported that a biodegradable coating based on *Malva neglecta* mucilage and CFS from the *Lactobacillus brevis* TD4 strain was effective in stabilizing the color of coated beef samples, resulting in the highest L*, a* and b* values at the end of the storage period compared to the control (El‐Mokhtar et al. 2020). In the study by Jooyandeh et al. (2023), a coating based on *Cordia myxa* mucilage and *Rosmarinus officinalis* essential oil effectively helped maintain the L* and b* color indices. Their findings also showed that although the a* values decreased during the storage period, this reduction was not statistically significant for the sample coated with 2% rosemary essential oil (Jooyandeh et al. 2023).

**FIGURE 6:**
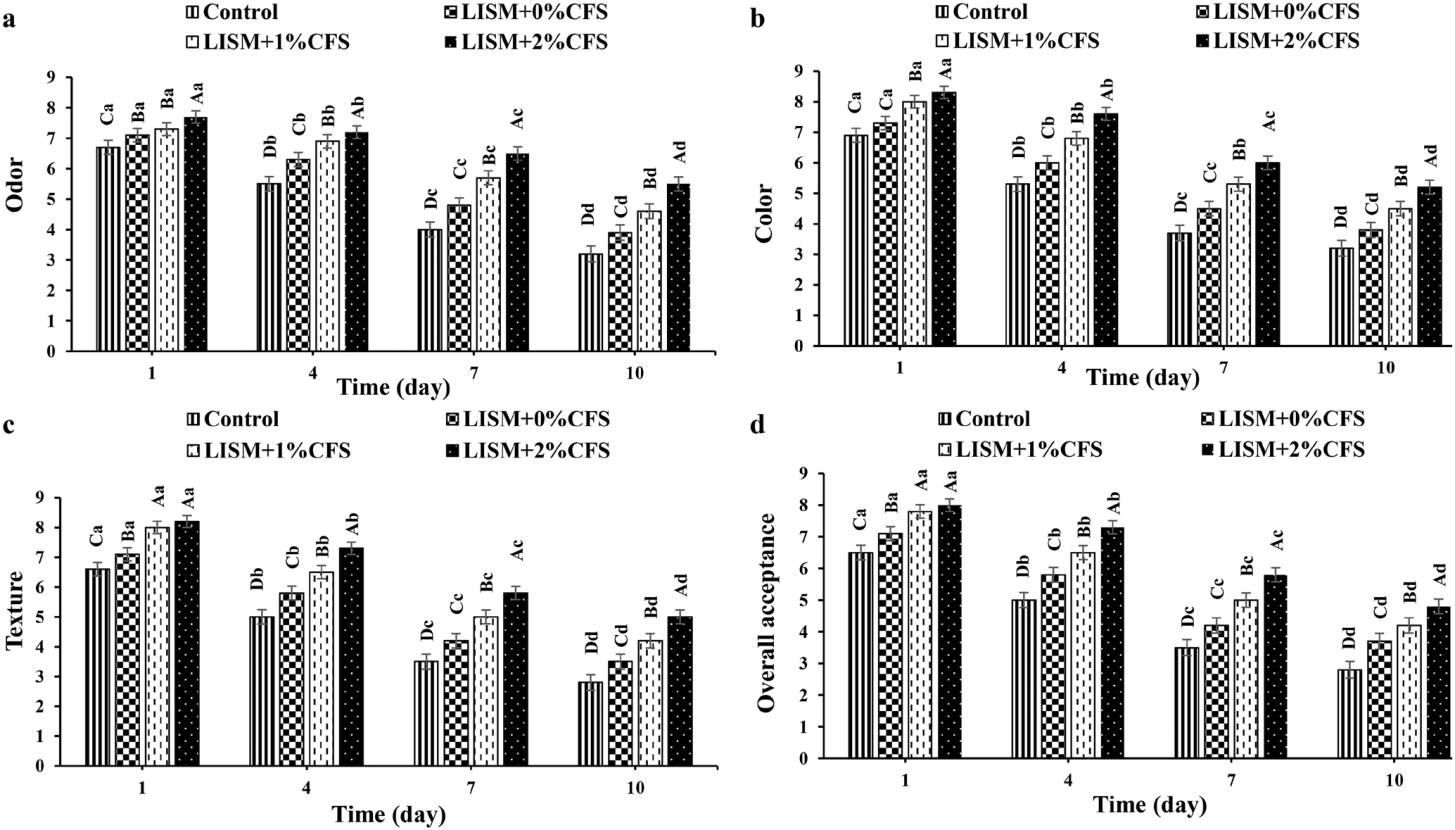
Changes in (a) odor, (b) color, (c) texture, and (d) overall acceptability of mutton samples coated with *Lallemantia iberica* seed mucilage (LISM) and different levels of cell‐free supernatant (CFS) of *Lactobacillus helveticus* strain C7303 during storage. Mean value ± standard deviation is presented. Capital letters indicate significant differences among treatments (A–D), and lowercase letters indicate significant differences across storage days (a–d) (*p* < 0.05). (*n* = 3 independent replicates, 52 panelists).

Sensory evaluation is one of the most important indicators in determining the final quality of a product and its acceptance by consumers, directly reflecting the physicochemical and microbiological changes of meat during the storage period. The results regarding the evaluation of sensory attributes, including odor, color, texture, and overall acceptance of the meat samples over the 10‐day period, are presented in Figure 6. In the nine‐point scale sensory evaluation, meat samples scoring 4 or higher are considered desirable and acceptable by consumers (Barzegar et al. 2020). Data analysis shows that the scores of all sensory parameters in all treatments exhibited a downward trend over time; however, the rate of quality decline in the coated samples was significantly slower than in the control sample. At the end of the storage period (day 10), the LISM+2%CFS treatment recorded the highest score in the odor parameter (5.50 ± 0.23), while this score for the control sample dropped sharply to 3.20 ± 0.26 (*p* < 0.05). A similar trend was observed in the color index, where the best performance belonged to the LISM+2%CFS treatment with a score of 5.20 ± 0.21 compared to the severe decline of the control sample (3.20 ± 0.26) (*p* < 0.05). In the texture parameter evaluation, the coating containing 2% active compound was able to maintain the score at an acceptable level of 5.00 ± 0.23, whereas the control sample had the lowest texture acceptance (2.80 ± 0.26) (*p* < 0.05). Finally, the examination of the overall acceptance index also showed that the LISM+2%CFS treatment significantly maintained its superiority over the control group at the end of the storage period and had higher acceptability (*p* < 0.05). The sensory evaluation results indicated a correlation between sensory attributes and microbial/physicochemical properties; such that samples with greater microbial and physicochemical stability received the highest sensory scores (Noshad et al. 2020). The findings of the present study are consistent with the results reported by Abbasi et al. (2024).

## 4 Conclusion

Biodegradable coating of mucilage of *Lallemantia iberica* and CFS of lactic acid *L. helveticus* strain C7303 with antimicrobial and antioxidant effects led to the prevention of oxygen transfer, reduction of the number of various microorganisms and increase the storage time of coated mutton samples. The mutton sample coated with mucilage +2% CFS had the lowest number of TVC, PTC, fungi and coliforms, the lowest values of pH, PV, TBA and TVB‐N parameters compared to the control sample. The results of the present study indicated that the mutton sample coated with mucilage +2% CFS had the highest moisture content, texture hardness, the highest amount of color parameters L*, a*, b* and the highest overall acceptance score. In general, the above biodegradable coating was able to increase the shelf life of the coated mutton samples at 4°C by preventing lipid oxidation, preventing the production of metmyoglobin and inhibiting tissue degradation.

## Author Contributions

**Alireza Vasiee:** writing – original draft, writing – review and editing. **Morteza Taki:** supervision, writing – review and editing, writing – original draft, software. **Behrooz Alizadeh Behbahani:** supervision, software, writing – original draft, writing – review and editing. **Hossein Jooyandeh:** writing – review and editing, writing – original draft. **Zeinab Mousavi:** investigation, writing – review and editing, writing – original draft, formal analysis.

## Funding

The authors have nothing to report.

## Ethics Statement

The sensory evaluation protocol of this study was reviewed and approved by the Research Ethics Committee of the Agricultural Sciences and Natural Resources University of Khuzestan, Iran. Prior to participation, the objectives and procedures of the study were fully explained to all assessors, and written informed consent was obtained from all participants. Participation in this study was entirely voluntary, and individuals were free to withdraw at any stage without any consequences. The confidentiality of information, anonymity of participants, and protection of data were strictly maintained throughout all stages of the research. All procedures were carried out with due consideration for the health, safety, and well‐being of the participants, and the collected data were used solely for scientific and research purposes.

## Consent

All authors have approved the manuscript for publication.

## Conflicts of Interest

The authors declare no conflicts of interest.

## Data Availability

The data that support the findings of this study are available on request from the corresponding author. The data are not publicly available due to privacy or ethical restrictions.
